# Quantitative expansion microscopy for the characterization of the spectrin periodic skeleton of axons using fluorescence microscopy

**DOI:** 10.1038/s41598-020-59856-w

**Published:** 2020-02-19

**Authors:** Gaby F. Martínez, Nahir G. Gazal, Gonzalo Quassollo, Alan M. Szalai, Esther Del Cid-Pellitero, Thomas M. Durcan, Edward A. Fon, Mariano Bisbal, Fernando D. Stefani, Nicolas Unsain

**Affiliations:** 1 Instituto de Investigación Médica Mercedes y Martín Ferreyra (INIMEC), Consejo Nacional de Investigaciones Científicas y Técnicas (CONICET), Universidad Nacional de Córdoba, Córdoba, Argentina; 2 Instituto Universitario Ciencias Biomédicas de Córdoba (IUCBC), Córdoba, Argentina; 3 Centro de Investigaciones en Bionanociencias (CIBION), Consejo Nacional de Investigaciones Científicas y Técnicas (CONICET), Buenos Aires, Argentina; 4 Departamento de Física, Facultad de Ciencias Exactas y Naturales, Universidad de Buenos Aires, Buenos Aires, Argentina; 5 McGill Parkinson Program, Neurodegenerative Diseases Group, Department of Neurology and Neurosurgery, Montreal Neurological Institute, McGill University, Montreal, Canada

**Keywords:** Biological techniques, Neuroscience

## Abstract

Fluorescent nanoscopy approaches have been used to characterize the periodic organization of actin, spectrin and associated proteins in neuronal axons and dendrites. This membrane-associated periodic skeleton (MPS) is conserved across animals, suggesting it is a fundamental component of neuronal extensions. The nanoscale architecture of the arrangement (190 nm) is below the resolution limit of conventional fluorescent microscopy. Fluorescent nanoscopy, on the other hand, requires costly equipment and special analysis routines, which remain inaccessible to most research groups. This report aims to resolve this issue by using protein-retention expansion microscopy (pro-ExM) to reveal the MPS of axons. ExM uses reagents and equipment that are readily accessible in most neurobiology laboratories. We first explore means to accurately estimate the expansion factors of protein structures within cells. We then describe the protocol that produces an expanded specimen that can be examined with any fluorescent microscopy allowing quantitative nanoscale characterization of the MPS. We validate ExM results by direct comparison to stimulated emission depletion (STED) nanoscopy. We conclude that ExM facilitates three-dimensional, multicolor and quantitative characterization of the MPS using accessible reagents and conventional fluorescent microscopes.

## Introduction

Fluorescence nanoscopy has revealed that actin, spectrin and associated proteins forming the cortical skeleton of axons and dendrites form annular structures which in turn are organized in a quasi-1-D periodic arrangement, with a characteristic spacing of ~190 nm^1,2^. The so-called actin/spectrin membrane-associated periodic skeleton (MPS) is composed of actin filaments organized in ring-like structures transverse to the axon and separated by various α/β-spectrin tetramers extended along the axon. This skeleton seems to be ubiquitously present in axons –and to a less extent in dendrites- and, more importantly, is a conserved structure of neurons from all animal species examined so far^3,4^. It has been proposed that the presence of the MPS is important in organizing membrane components^5^, regulating axon diameter^6^, controlling microtubule dynamics^7^, and axon fragmentation during developmental pruning^8^. A deeper knowledge of this structure is likely to provide new insights for a more complete understanding of axon biology.

The characteristic feature of the MPS (the one-dimensional (1-D) periodic distribution of its components) is below the resolution limit of conventional microscopy -due to the diffraction limit of light. Thus, its study has been governed by the use of a handful of high-end super-resolution microscopy approaches, often referred to as fluorescence nanoscopy, including stochastic optical reconstruction microscopy (STORM), stimulated emission depletion (STED) and super-resolution structured illumination (SR-SIM). These techniques require specialized equipment, sophisticated data analysis and dedicated trained users^9,10^, meaning their application is far from widespread in biomedical research laboratories. This report explores the possibility of using expansion microscopy (ExM) to solve this issue, making it simpler for neurobiology research groups to study this intriguing structure.

In the early 80’s, Tanaka and colleagues rigorously described that a polymer network of partially ionized acrylamide can undergo as many as 100-fold discrete reversible volume transitions^11,12^. These observations gave rise to the promising technology of “tunable” and “intelligent” phase-transition gels that would trap and deliver cargoes for specific purposes, that include medical and industrial applications^13^. In 2015, Boyden and colleagues smartly used the swelling behavior of hydrogels to physically expand preserved biological specimens, which introduced the field of *Expansion Microscopy*^14^. Briefly, their protein-retention approach (pro-ExM) version of the technique consisted of polymerizing a 2.5% acrylamide gel (ionized with sodium acrylate) within a cell and cross-linking endogenous proteins to the gel. Protein digestion was next performed to ensure isotropic expansion when the specimen’s proteins were pulled apart during expansion of the hydrogel by water dialysis. Relative protein location was preserved and meaningful information about protein distribution in cells and tissues (labeled before or after expansion) was obtained by conventional fluorescent microscopy. The empirical resolution of the re-scaled image, consequently, improves linearly with the expansion factor obtained. On top of increasing resolution, ExM is also convenient for imaging thick specimens, since expanded samples are almost transparent given that their composition is ~99% water.

In ExM, to extract quantitative information from expanded samples, it is imperative to have a correct estimation of the expansion factor at which the cellular components were pulled apart. So far, expansion factor estimations have been performed either by measuring the gel block before and after water dialysis-induced swelling^15,16^, by measuring the very same location before and after expansion^17,18^ or by studying structures whose dimensions were already known by other techniques, such as fluorescence nanoscopy or electron microscopy. The values reported by these approaches vary significantly, with macroscopic measurement of the gel block seeming to underestimate the actual expansion of cellular proteins. Here we explored the possibility that the hydrogel built within the dense protein meshwork of the cell swells more than the hydrogel that lies outside the cells (the gel block).

Our corrected estimation of expansion factors enabled us to perform the quantitative characterization of the MPS. Because ExM involves various steps of chemical manipulations, including cross-linking reactions, protein digestion, and dialysis, by which the original disposition of the antigens may be distorted or lost, it is advisable to validate the parameters of an ExM protocol for a specific sample or cellular structure^19^. Here, we optimized, calibrated and validated the use of ExM for the nanoscale characterization of spectrin MPS of axons in culture. Our resulting protocol uses a combination of steps and parameters previously validated in separate works. We show that, to accurately estimate pre-expansion dimensions, expansion coefficients have to be calculated using large intra-cellular protein structures. We further validate the protocol by comparing the abundance and organization of MPS obtained by ExM with those obtained using STED microscopy. We conclude that properly calibrated ExM is a straightforward, multicolor, three-dimensional approach to study the MPS that can be rapidly incorporated in neurobiology laboratories.

## Results

In expansion microscopy of cultured cells, correctly estimating the dimensions of cell structures depends on precisely estimating the expansion factor at which these structures were pulled apart. The swelling of the gel block is commonly used as a prompt to estimate the expansion factor of the label and thus the “real” -pre-expansion- dimension of the protein structures under study^15,16^. However, since the cell is a dense aggregate of proteins and these are cross-linked to the acrylamide polymer that is made within the cell, we speculated that the swelling ratios of this ionic polymer and those of the ionic polymer densely cross-linked to cellular proteins would differ. Another factor to consider is that these proteins cross-linked to the hydrogel are partially digested by incubation with the protease proteinase K.

**Figure 1:**
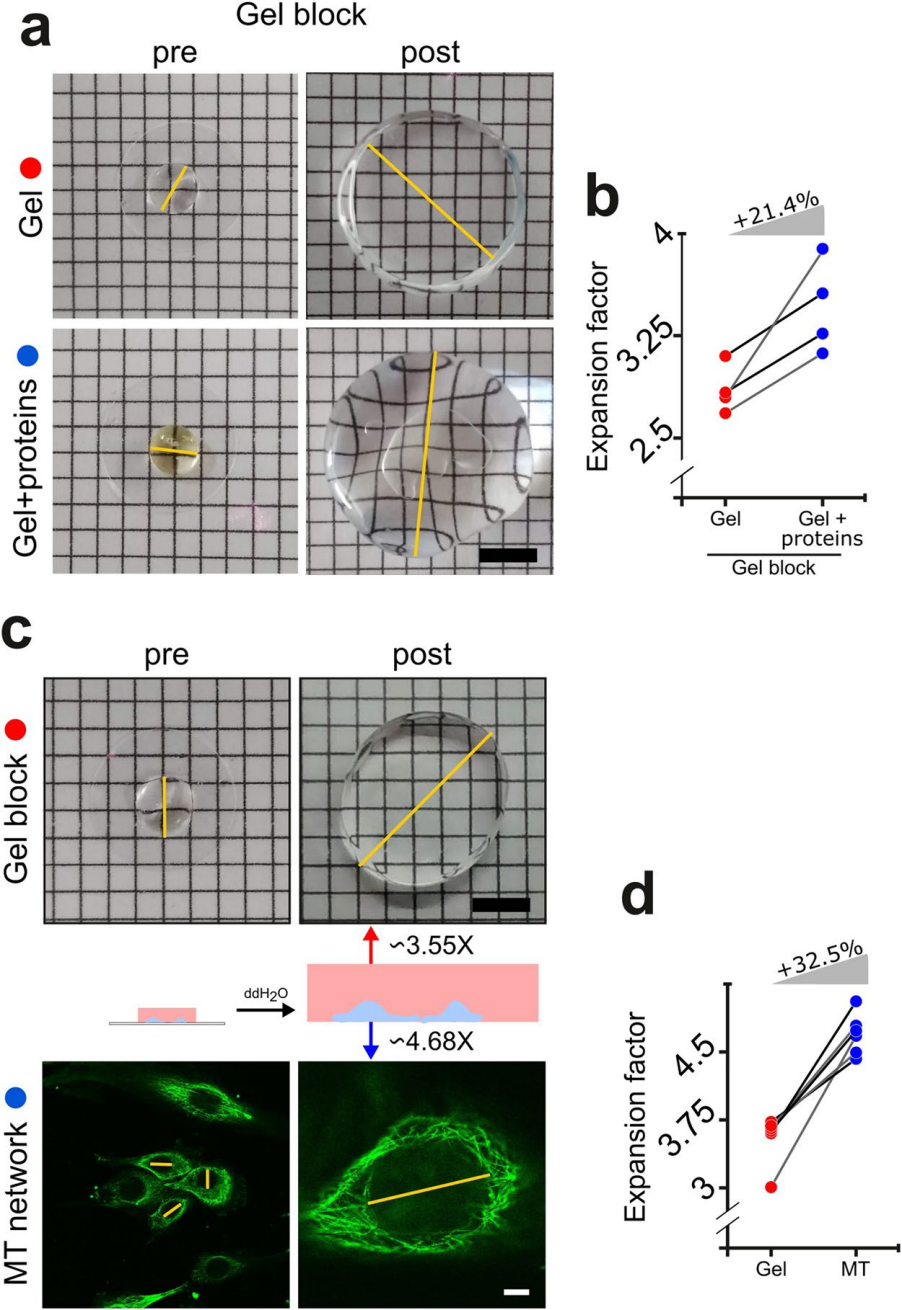
Calibration of *expansion factors* for a correct estimation of pre-expansion dimensions. (**a**) Representative images of regular gels (top) or gels that had proteins dissolved in the monomeric solution (bottom), before and after expansion. The yellow lines exemplify the measurements of the longest diameter performed to calculate expansion factors. Scale bar: 5 mm. (**b**) Gels prepared with a monomeric solution that had proteins dissolved expanded ~21.4% more the gels prepared with the same monomeric solution without proteins. Paired red and blue circles correspond to the same experiment, which uses the same starting monomeric solution. (**c**) Images of a regular ExM gel block (top) and widefield images of NIH-3T3 cells stained for alpha-tubulin (bottom) before (left) and after (right) expansion. The yellow lines exemplify the measurements of the longest diameter performed to calculate expansion factors. The scheme in the middle depicts the differential expansion that take place in different objects of the same experiment. Scale bars: 5 mm (gel block) and 15 µm (cells). (**d**) When the gel block factor is compared with the corresponding microtubular network factor inside that same gel (paired red and blue circles), a consistent ~32.5% increase in expansion factor is found across experiments. We show the mean values from 6 different experiments and at least 3 gels and 40 cells were measured in each experiment before or after expansion to obtain the corresponding expansion factors.

As a first test of our hypothesis, we compared the effective expansion of routine acrylamide gels used for expansion microscopy against the same recipe but containing yeast proteins that were dissolved in water and cross-linked before gelation. Interestingly, gels with proteins expanded significantly more (~21.4%) than gels alone (Fig. 1a,b). As a next step we investigated whether this finding was relevant for expansion microscopy experiments. We chose to evaluate the expansion factor of the network of microtubules in cultured fibroblasts and compared this with the expansion of the gel block (i.e. the gel that polymerized outside the cells and makes up the macroscopic gel block). Microtubules arising from the centrosome in these cells delineate the nucleus, and the longest diameter of this easy-to-locate topographic feature provides a population measurement to compare before and after expansion. The expansion factor of this structure of proteins within the cell (Ex-factor-Proteins) was substantially larger than the expansion factor of the gel block containing the cells (Ex-factor-Gel, Fig. 1c,d). Specifically, while the Ex-factor-Gel was 3.55 ± 0.25, the Ex-factor-Protein was 4.68 ± 0.21 (n = 6, mean ± SD, Fig. 1d). We estimated a lateral resolution of 360.51 ± 67.09 nm (n = 30, mean ± SD) for our microscope (when using an objective of NA = 1.49) by measuring the full width at half maximum (FWHM) of images of fluorescent beads (λ_emission_ = 510 nm) with a diameter of 20 nm (*data not shown*). Therefore, after expansion we expect a lateral resolution of 77.03 nm (360.51 nm/4.68).

**Figure 2:**
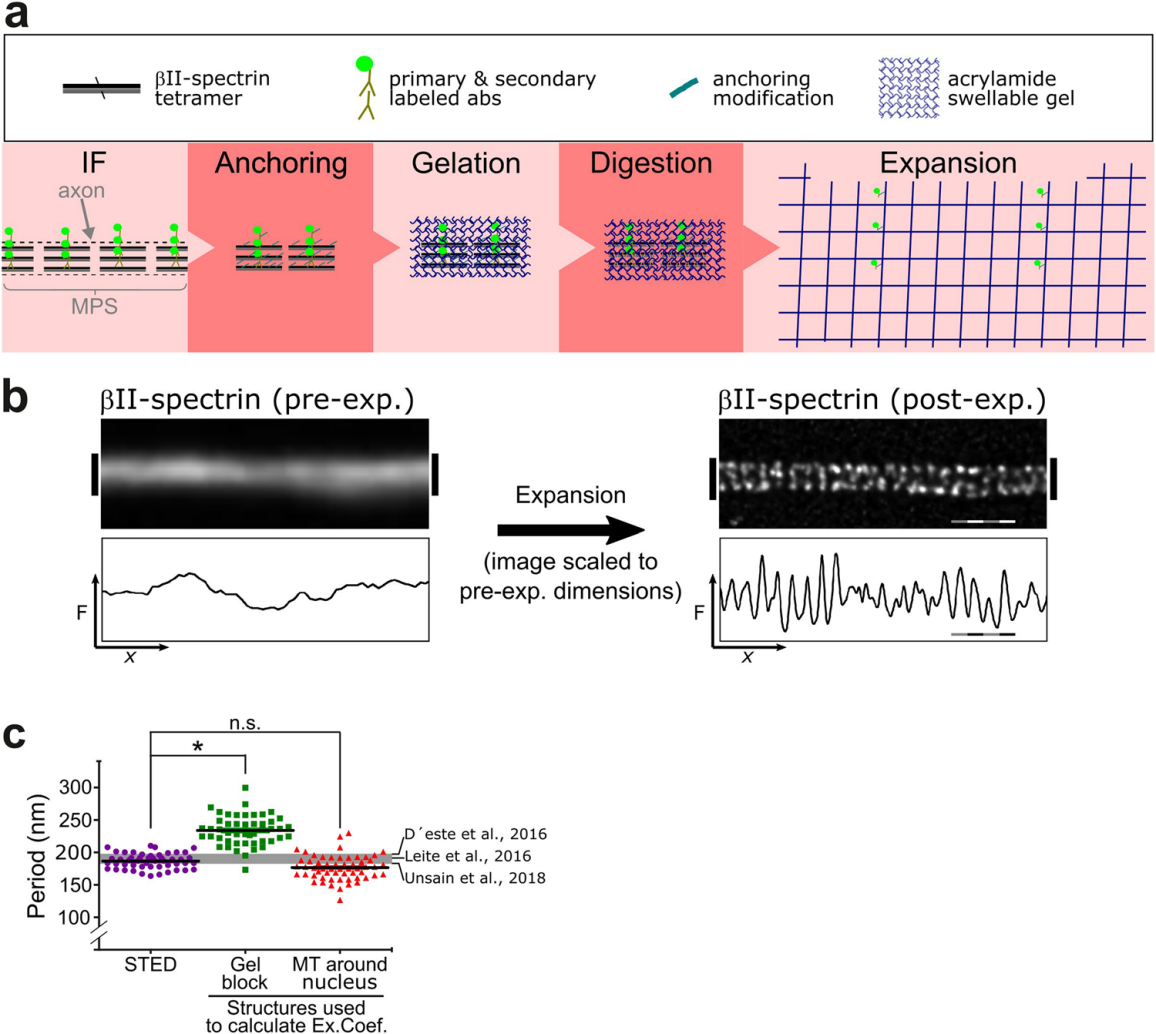
Expansion microscopy reveals the periodic distribution of βII-spectrin in axons. (**a**) Scheme summarizing the different steps of the ExM protocol used for axonal βII-spectrin. (**b**) The immunofluorescence against βII-spectrin shows no evident periodicity in its distribution before expansion (left). The trace below is an intensity profile across the image shown. After expansion, the fluorescent-labeled antibodies disperse ~4 times in each dimension and the images obtained are scaled to depict pre-expansion dimensions (right), the signal shows the expected periodicity at ~190 nm. Scale bar: 760 nm, subdivided in 4, 190 nm segments. (**c**) Periodicity values in DRG sensory axons obtained by STED, and expansion microscopy. Expansion microscopy pre-expansion dimensions were calculated using the factor obtained by measuring the gel block or the microtubule network around nuclei. The gray line at 190 nm summarizes the values of periods obtained before for sensory neurons, as cited in the figure. We measured 30 periods per group, in 1 µm segments.

In order to validate our approach for quantitative expansion microscopy we applied it to the visualization of the periodic organization of βII-spectrin in the MPS of axons and dendrites. Our expected resolution of 77.03 nm is sufficient to clearly detect the periodic structure of the MPS, which has a period of 190 nm. Explants of dorsal root ganglion (DRG) sensory neurons cultured for 3 days *in vitro* (DIV) were stained for βII-spectrin with a monoclonal antibody against the N-terminus of the protein (near the tetramerization site of the αII/βII-spectrin tetramer), followed by a secondary antibody labeled with the fluorophore Alexa Fluor 488. Non-expanded axons showed a βII-spectrin signal with no evident periodicity under the wide field microscope. When inspected with STED nanoscopy, the characteristic periodic structure of βII-spectrin in the MPS was clearly visible, with a mean distance between peaks of 197.7 ± 10.61 (mean ± SD, Fig. 2c, purple circles), in agreement with previous reports^6,8,20^. Analyzing STED images of the MPS obtained at different depletion powers (and thus different resolutions) we determined that the minimum lateral resolution necessary to detect the periodic distribution of βII-spectrin in our samples is of 115 nm (See Supplementary Fig. S1 for further details).

For pro-ExM, we tested different variables from the original protocol^14^ and subsequent modifications^17,18^ and, in our hands, 2-hours gelation at room temperature, 15-minutes glutaraldehyde crosslinking (“anchoring”), and proteinase K digestion overnight at room temperature gave the best balance of simplicity, speed and consistency (Fig. 2a). In expanded samples, the periodic distribution of the βII-spectrin signal in the axonal MPS is readily visible (Fig. 2b). The expansion of βII-spectrin immunolabeling was also effectively achieved in cell somas and dendrites (Supplementary Fig. S3), yielding similar patterns of staining as previously reported^21^. A quantitative assessment of the observed structures depends directly on the accuracy of the expansion factor. If the expansion factor determined from the expansion of the gel block (Ex-factor-Gel) was used, as is commonly done in ExM, a significantly overestimated βII-spectrin period of 240.6 ± 20.69 nm (mean ± SD) was obtained (Fig. 2c, green squares). Remarkably, if the Ex-factor-Protein is used, obtained from gels made in the same run with the same monomeric solution, we obtained the correct period of βII-spectrin (Fig. 2c, red triangles, 188.9 ± 16.96 nm, mean ± SD). These results indicate that determining the expansion factor using cellular protein structures such as the microtubule network is a suitable and simple approach to achieve quantitative expansion microscopy.

To further validate this approach, we interrogated the fidelity of expansion using the 190 nm periodicity of the MPS as a standard ruler. First, we analyzed consistency within a region of interest (ROI) of ~110 × 110 µm, and found that the difference among the mean periods measured in any sub-region within this ROI is below 5% and not statistically significant (Supplementary Fig. S2). We then tested consistency across longer distances within the same gel. The initial gel is 5 mm wide. After expansion, we selected 4 ROIs of 2500 µm^2^, ~8 mm apart, and found that all determinations of the βII-spectrin period fall within the expected dispersion of the MPS and that the largest difference between sites was below 5% (Supplementary Fig. S2). We therefore conclude that expansion microscopy is a quantitative super-resolution imaging tool when the expansion factor is determined using cellular protein structures such as the microtubule network.

**Figure 3:**
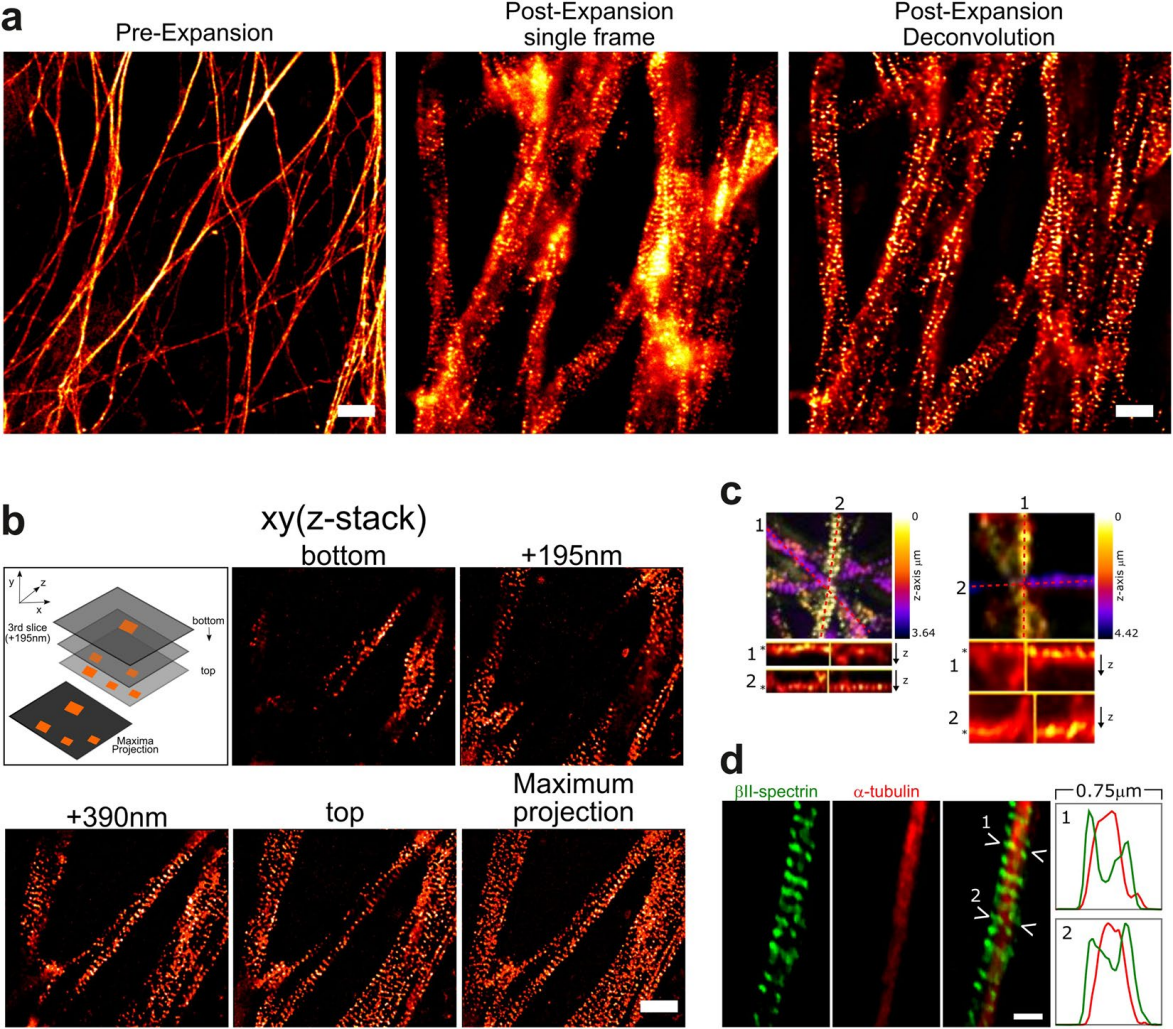
ExM allows three-dimensional and multicolor examination of the MPS. (**a**) Representative widefield images of βII-spectrin immunofluorescence before (left panel) and after expansion (middle and right panels). The middle image is a single widefield frame out of a stack made of consecutive steps in the z-axis. The right image is the corresponding frame after deconvolution processing, to eliminate out-of-focus light. Scale bars: pre-expansion 4 µm and post-expansion 1 µm. (**b**) Selected images from a z-stack, after deconvolution. The last panel shows a maximum projection of the shown stack. Scale bar: 1 µm. (**c**) x/y maximum projections of selected z-stacks are shown and color-coded for depth. The bottom panels show x/z sections #1 and #2. Asterisks indicate the depths at which crossing axons with an MPS are visible. Scale bar: 1 µm. (**d**) Transversal intensity profiles of double-labeled axons show the expected peripheral staining of βII-spectrin compared to the more inner staining of microtubules stained with an antibody against tyrosinated tubulin (profiles #1, and #2). Scale bar: 0.5 µm.

Further, we explored the use of quantitative expansion microscopy for three-dimensional (3D) visualization of the MPS. We examined the expanded samples in several planes in the axial coordinate (Fig. 3a). The contrast of each single-plane image was improved using a deconvolution algorithm to eliminate out-of-focus light (Fig. 3a). Easy-to-obtain 3D images are especially advantageous in cultures with dense axonal fields, as in the case of sensory explants (Fig. 3b) or long-term cultures. Another advantage of the gain in resolution in three dimensions is the possibility to discern the MPS from multiple axons in crowded regions of interest (Fig. 3c), which are normally avoided when using the most common 2D nanoscopy approaches. All these make this approach a simple and straightforward three-dimensional, super-resolution technique.

We then confirmed that ExM for the MPS allows simple dual-color imaging. Since the technique is compatible with regular immunofluorescence protocols and most commercially available fluorophores^19^, the number of simultaneous detections is limited only by the excitation/emission capabilities of the optical system being used. For example, double detection of the MPS (anti-βII-spectrin) and microtubules (anti-α-tubulin) can readily show the differential localization of these molecules in the *xy* plane, combining multiple axial optical sections (Fig. 3d and Supplementary Fig. S4). The resolution achieved did not allow the visualization of individual microtubules in thin axons, but allowed distinguishing them in cell somas and in thick neurites (Supplementary Fig. S4).

**Figure 4:**
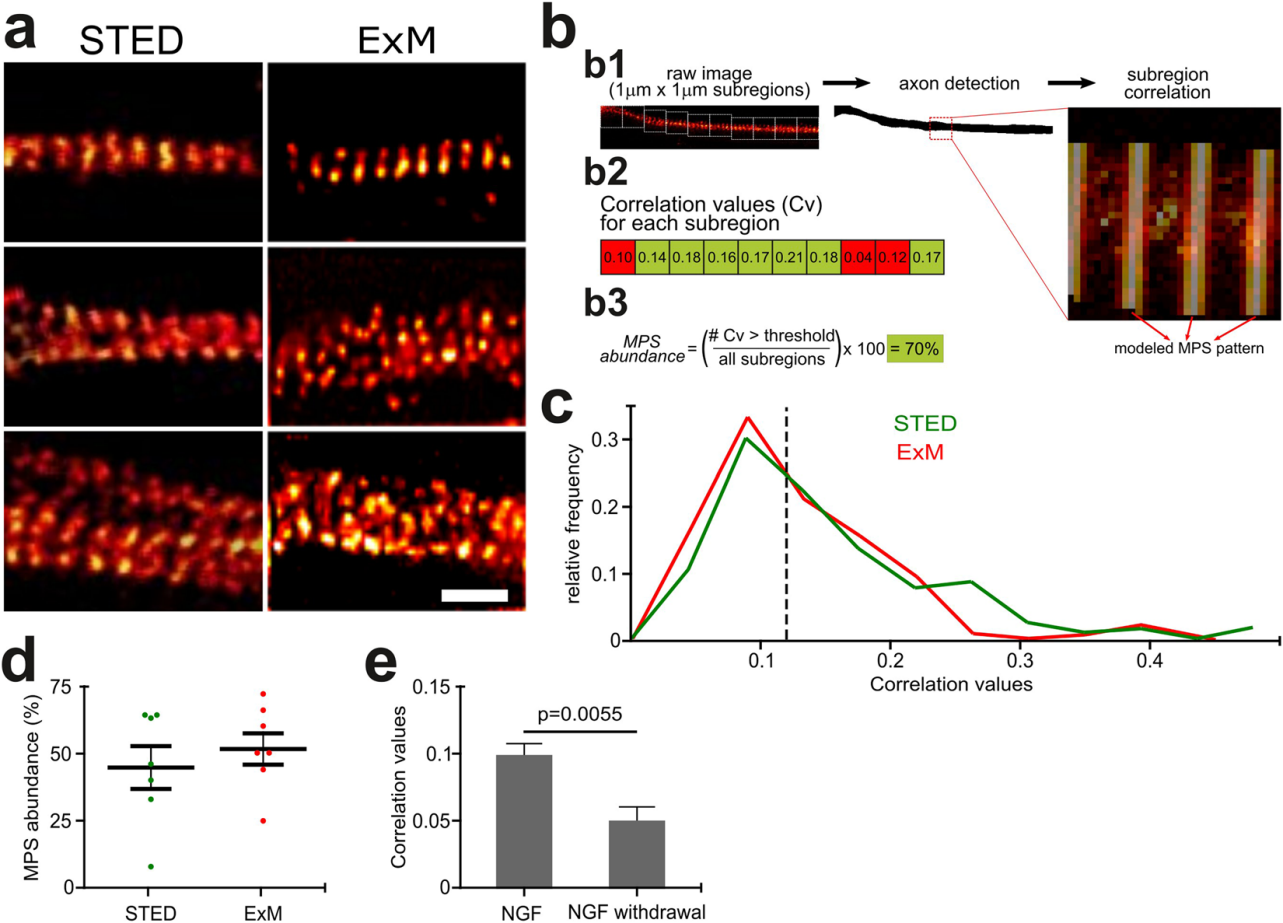
Quantitative validation of organization of the MPS in sensory axons as obtained by ExM compared to STED nanoscopy. (**a**) Selected images from βII-spectrin stained DRG sensory axons obtained by either STED or ExM. Scale bar: 500 nm. (**b**) The Gollum software enabled quantitative assessment of the global *correlation values* (as shown in (**c**)) and estimation of the *abundance* (as shown in **d**). Briefly, the algorithm first recognizes axons and lays consecutive 1 µm × 1 µm segments. A modeled MPS pattern is superimposed on the image (b1) and a Pearson correlation is obtained for each segment (b2). *MPS abundance* is calculated by the proportion of segments with a correlation value above a defined threshold correlation that would correspond to a visible MPS, as predefined by the user (b3). See *Materials and methods* for more details. (**c**) Histogram showing the global correlation values obtained by STED (green line, n = 300) compared to ExM (red line, n = 140). (**d**) Graph showing the MPS abundance as observed by STED or ExM. Mean ± SEM, n = 7. (**e**) Mean correlation values obtained from control sensory axons (NGF), compared to axons subjected to NGF withdrawal for 12 hours. Mean ± SEM. n = 20.

The anchoring, protein digestion and pulling apart of protein structures present in any ExM protocol may produce artifacts in highly-ordered protein structures such as the MPS that may preclude its use to study its native arrangement. Hence, to discard the presence of such artifacts, the distribution and organization of the MPS in 3 DIV DRG axons was compared quantitatively between samples examined by ExM and by a commercially available STED nanoscope (Abberior GmbH, Germany). The fluorophores linked to the secondary antibody varied according to the system requirements: Atto-647N was used for STED and Alexa Fluor 488 for ExM. Atto-647N also works for ExM, but we preferred to use smaller wavelengths for the increased gain in resolution associated with smaller wavelengths. To the eye, both approaches uncovered similar periodic structures in axons of comparable width (Fig. 4a). The regularity of the MPS along axons was determined using *Gollum*^22^, an open-source image-analysis software recently developed by our group for quantitatively assessing the regularity of periodic structures. The algorithm calculates Pearson’s correlation of axonal segments against a predefined modeled MPS (Fig. 4b). Using this approach, we first evaluated the regularity of the MPS in equivalent axonal segments, finding no significant difference between samples examined by ExM or STED (Fig. 4c), which was also found when we compared the abundance of MPS with each procedure (i.e. proportion of 1 μm × 1 μm segments showing a correlation consistent with a periodic distribution, Fig. 4d). These results show that the observation of the MPS by ExM yields results that are indistinguishable from routine STED nanoscopy.

It was recently reported by STED and STORM that the MPS suffers significant remodeling prior to axon fragmentation in a model of developmental axon pruning^8,23^. After 3 days of growth in culture, dorsal root ganglion explants were deprived of nerve growth factor (NGF withdrawal), which triggers axon degeneration, culminating 24 hours later in axon beading and fragmentation. By ExM of βII-spectrin, we were able to confirm the extent of remodeling previously reported, at 12 hours of NGF withdrawal (Fig. 4e).

## Discussion

We show the optimization, calibration and validation of an expansion microscopy protocol for examining the spectrin membrane-associated periodic skeleton, which previously had only been visualized with fluorescence nanoscopy methods. Since its first publication by Edward Boyden and colleagues^17^, numerous protein-retention expansion microscopy protocols have been reported, each improving some aspects of the original protocol, for example, to tackle a specific cellular structure or a challenging sample, to improve antibody retention or to preserve fluorescence intensity after expansion^18,24–28^. We present a simple approach that will allow the quantitative study of the MPS in cultured neurons using conventional microscopes, which we believe may be very useful for neurobiology groups aiming to contribute to the understanding of this intriguing structure.

We first had to accurately calculate the expansion of the specimen to unequivocally determine the pre-expansion distances. We noticed that the commonly used gel expansion measurement underestimates the actual expansion of protein structures within the cell. The swelling and rheological properties of hydrogels have been extensively studied, due to the interest in their application in the design of “intelligent” materials that can trap or deliver cargoes under specific changes in environmental properties or ligands^13,29^. It has been shown that hybrid hydrogels containing specific recombinant proteins significantly modify the swelling and the physical properties of the composed hydrogel^30–33^. Taking this into account, it is not surprising that the hydrogel within a cell can be considered a protein-containing hybrid hydrogel and we show that, in the conditions of our experiments (i.e. hydrogel composition, gel-protein cross-linking, protein digestion by proteinase K, and swelling at room temperature by water hydration), the net effect is that the hydrogel-protein mix swell to a greater extent than the surrounding hydrogel devoid of proteins (i.e. outside of the cells). Hydrogels will expand to a maximum that will reflect a balance between the solvation of polymer chains and the elastic force exerted by the polymer network immobilized through cross-links. When a great diversity of proteins at relatively high concentrations is added to the mix, the balance between these two forces will shift, ultimately changing the equilibrium volume reached. This change can be due to changes in the polymer-solvent interaction parameter, disruption/formation of crosslinks, or order-disorder transition of polymer domains. To take this into account, we measured the microtubule network delineating the nucleus before and after expansion in cultured cells performed side-by-side with the axonal preparations. This is an accessible approach that will allow pre-expansion measurements to be accurately established. End users may find other protein structures more convenient for their specific setting, but such a structure should fulfill certain conditions: its non-expanded dimensions should be non-diffraction limited, it has to be easily recognized both pre- and post-expansion, it should be reasonably invariant in size to simplify the sampling effort, and all this must be performed with the same reagents as the experiment being performed.

This quantitative approximation of expansion factor estimations is useful when studying cellular structures of unknown dimensions or when small size changes can bear relevant biological information that would otherwise be washed out by an inaccurate estimation of expansion factors.

Because the MPS is a highly-ordered and compact multi-protein structure that may suffer distortion or loss in different portions of the axon during the steps involved in ExM, we further validated our observations by comparing our quantitative measurements with those obtained with STED nanoscopy, a widely used super-resolution approach. We found that the quantitative estimations of the organization and abundance of the MPS in sensory axons are indistinguishable between the correctly calibrated ExM and STED. Moreover, ExM was able to show the remodeling of the MPS during axon degeneration emulating developmental pruning, which was first reported using STED^8^ and later confirmed by others using STORM^23^.

Most STED and STORM set-ups provide roughly three times more resolution than the one obtained with regular pro-ExM. However, to study the prominent features of the MPS in large axon populations, we believe the ExM protocol proposed here has considerable advantages. First, image acquisition is as fast and straightforward as in any conventional fluorescence microscope and large areas of interest can be examined in short times. Also, the same region can be imaged repeated times before significant photobleaching occurs. Second, while each fluorescence nanoscopy method works well only with a restricted set of fluorophores, pro-ExM can be applied with a considerably larger set of fluorescent labels^17^. Third, three-dimensional acquisitions, which are of special interest when examining samples with crowded axon populations, is considerably simpler than in any fluorescence nanoscopy implementation.

In summary, we have optimized, calibrated and validated a quantitative expansion microscopy approach for the examination of the MPS that will allow the broad neurobiology community to include the observation of this conserved structure in their studies and contribute to our understanding of its function in axon biology and pathophysiology. Furthermore, it is sensible to expect that our protocol will also be useful for the investigation of any other sub-cellular structure in neurons, and probably other cell types.

## Methods

### Antibodies

Mouse anti-βII-spectrin was from BD Biosciences (US, cat. #612563) and used 1:100. Rat anti-tyrosinated tubulin was from Abcam (cat. #ab6160) and used 1:200. Mouse anti-alpha tubulin was from Sigma-Aldrich (clone α-3A1, cat. #T5168) and used 1:400. The corresponding secondary antibodies for widefield were Alexa Fluor 488 and 568 (Thermo Fisher Scientific, anti-mouse-AF488 cat. # A32723, anti-rat-AF568 cat. #A11077); and anti-mouse ATTO-647N was used for STED (Sigma-Aldrich, cat. #50185).

### Animals

Time-pregnant C57Bl/6 mice and Wistar rats were provided by our Specific Pathogen Free Animal Facility. The lines were originally provided by Laboratorio de Animales de Experimentación (LAE, Facultad de Ciencias Veterinarias, Universidad Nacional de La Plata, Buenos Aires). All procedures involving animals were approved by the institution’s Council of Animal Care (INIMEC-CONICET, Reference number: Res 006/2017 A), by the National Department of Animal Care and Health (SENASA, Argentina) and were in compliance with the general guidelines of the National Institute of Health (NIH, USA). Efforts were made to minimize the number of manipulations and the animals used. Time-pregnancies were achieved in animals from 2 to 6 months of age. Animals were under standard cages, with free access to food and water, with a 12-hour light/dark cycle and controlled humidity and temperature (40–70% and 20–26 °C, respectively).

### Cell cultures

#### Sensory explants

Dorsal root ganglion (DRG) explants were prepared from E13.5 C57Bl/6 mouse embryos and grown on 12 mm cell culture coverslips (Marienfeld Superior; Cat. #633029), coated sequentially with (poly)ethyleneimine polymer (PEI) (2%, Sigma-Aldrich), laminin (10 mg/ml; Sigma-Aldrich), and collagen (0.1 mg/ml, PureCol; Advance BioMatrix). The culture media consisted of Neurobasal (Thermo Fisher Scientific; Cat. #21103049) supplemented with 2% B-27 (Thermo Fisher Scientific, Cat. #A3582801), 1% L-glutamine (Wisent), 1% Penicillin‐Streptomycin (Thermo Fisher Scientific, Cat. #15140122) and Nerve Growth Factor (NGF, 50 ng/ml, Harlan). NGF withdrawal for degeneration experiments was performed by changing media at 3 DIV with a media lacking added NGF, and fixed 12 hours later.

#### Hippocampal cultures

Hippocampal cultures were prepared as described previously^34^. Briefly, Wistar pregnant rats were sacrificed by CO_2_ asphyxiation under isoflurane anesthesia and hippocampi from E18 embryos were dissected and treated with trypsin (0.25% for 15 min at 37 °C, Thermo Fisher Scientific Fisher Gibco, Cat. #15090‐046) and mechanically dissociated by trituration with a Pasteur pipette. Cells were plated on 12 mm cell culture coverslips coated with 1 mg/ml poly‐L‐lysine (Sigma-Aldrich, Cat. #P2636) at a density of 2000 cells/cm^2^ in Minimum Essential Medium (MEM, Thermo Fisher Scientific, Cat. #61100‐061) supplemented with 1% Penicillin‐Streptomycin, 1% GlutaMAX I Supplement (Thermo Fisher Scientific, Cat.# A1286001), sodium piruvate (Thermo Fisher Scientific, Cat. #11360070) and 10% horse serum (Thermo Fisher Scientific, Cat. #16050122). After 2 h, the coverslips were transferred to dishes containing serum‐free Neurobasal media, supplemented with 2% B-27, 1% GlutaMAX I Supplement and 1% Penicillin‐Streptomycin.

#### NIH-3T3 cell line

Frozen vials were recovered and then maintained in Minimum Essential Medium supplemented with 10% fetal bovine serum (Thermo Fisher Scientific, Cat. #16050122). Cells were plated on 12-mm circular coverslips and used at 80% confluence.

### Immunocytochemistry

Samples were fixed with a solution containing 4% paraformaldehyde and 4% sucrose in PBS for 20 min at RT, permeabilized with PBS-Triton 0.2% for 5 minutes and blocked with two incubations of 5 minutes with PBS-Tween 0.1%. Primary antibodies were incubated overnight at 4 °C and secondary antibodies were incubated for 1–2 hours at RT.

### Sample preparation for expansion microscopy

#### Anchoring

For anchoring, the cells were incubated in glutaraldehyde 0.25% diluted in PBS during 10 minutes at RT (as described elsewere^18^).

#### Gelation

Monomer solution stocks (stored at −20 °C, sodium acrylate, 8.6 mg/ml, acrylamide, 2.5 mg/ml, N, N’-methylenebisacrylamide, 0.15 mg/ml, sodium chloride, 11.7 mg/ml; PBS 1 × ; ddH_2_O) were thawed and kept at 4 °C. A drop of monomer solution was placed on top of cells/explants for 5 minutes. TEMED (tetramethylethylenediamine, accelerator) and APS (ammonium persulfate, initiator) were added to the monomer solution up to 0.2% (w/w) each, both from a 10% stock solution in ddH_2_O and poured into a 5 mm diameter silicone (Sylgard) well. The coverslip with cells/explants was placed upside-down on top, making sure to seal the well. Gelation was allowed to occur for 2–3 hours at RT.

#### Digestion

The coverslip with the attached gel was put on a 12-well plate and incubated with proteinase-K (8 units/ml) diluted in digestion buffer (Tris-Buffer 50 mM, pH = 8.5, Biopack; EDTA 1 mM, Invitrogen; Triton X-100 0.5%; guanidine-HCl 0.8 M, Sigma) overnight at RT.

#### Expansion

Following digestion, dialysis with ddH_2_O was performed. Fresh ddH_2_O was exchanged 4–5 times every 2 hours until the expansion of the gel plateaued. The gels were expanded in 6-well plates.

#### Gel + proteins experiment

For the experiments in Fig. 1a,b, the gels were prepared as follows: the gel solution was prepared as explained before, with the addition of a portion of water containing dissolved yeast proteins (i.e. 94 µl of monomer solution, 2 µl of 10% TEMED, 2 µl of 10% APS and 4 µl of ddH2O or 4 µl of the protein solution). The yeast protein extract (Britania, cat. #B0100606) was dissolved in ddH2O (46% w/v) and was incubated with glutaraldehyde (0.25% w/w, anchoring step) for 1 h, RT, shaken and covered from light). The following steps (gelation, digestion and expansion) were performed as explained above.

### Expansion microscopy imaging and processing

Post-expansion images were captured using an Olympus IX81 inverted microscope equipped with a disc rotation unit (DSU), and either a 60x (NA: 1.42) or a 100x (AN: 1.40) oil objectives on the epifluorescence illumination (150 W xenon) lamp and a microprocessor. The gels were sampled in z-axis of 260 nm steps to get a z-stack, at an exposure time of 400–800 ms. For the protein expansion coefficient measure, the images were captured with confocal microscopy Olympus FV1000 with a 60x objective. To observe the gels under the microscope, they were placed in a glass-bottom 35 mm culture dish and all excess water was eliminated to avoid gel drift during image acquisition. Note that all dimensions of expanded samples in this report have been divided by their respective measured expansion factor and thus refer to pre-expansion dimensions (unless otherwise specified). All quantitative analyses were performed on the raw images, and deconvoluted images were used only for the purpose of rendering.

#### Deconvolution

The ExM images were deconvoluted using the FIJI software^35^. Images were processed with the Richardson-Lucy deconvolution algorithm with TV regularization and 100 iterations (Deconvolution Lab plugins).

#### Z-stack color coding

The z-stack of deconvoluted images was processed using the *Temporal-Color Code (Fire look-up-table)* macro in FIJI software. As a result, a 2D image with a color code of locations in the z-plane was obtained.

### Expansion factor calculation

#### Expansion factor from the Gel

For each experiment, we took a picture of the gel pre- and post-expansion on top of a ruler. Using the FIJI software, gel diameters were measured pre- and post-expansion. Pre-expansion and post-expansion diameters were averaged and then divided to obtain the expansion factor (Ex-factor-Gel).

#### Expansion factor from the microtubular network around nucleus

The longest diameter of the microtubular network around nuclei was measured in ~40 NIH-3T3 cells pre- and post-expansion. The population values were averaged and then divided (post/pre) to estimate the corresponding expansion factor (Ex. factor-MT). Microtubules were stained with an antibody against alpha-tubulin.

### STED nanoscopy

Stimulated Emission Depletion nanoscopy (STED) was performed on two nanoscopes: 1) STED Quad Scan super-resolution microscope (Abberior Instruments, Germany), installed on an Olympus IX 83 inverted microscope (Microscopy Imaging Center, Montreal Neurological Institute, McGill University). Samples were excited with a 635 nm laser, and depleted with a 775 nm laser. Images were obtained at a single plane, and with the pinhole open to ~1 Airy unit (more details on STED acquisitions using this instrument can be found in the supplier´s website). 2) A custom-built nanoscope at the Center for Bionanoscience Research (CIBION, CONICET) for which a detailed description was published before^22^. Briefly, samples were excited with a linearly polarized pulsed (200 ps) laser at 640 nm (PicoQuant LDH-P-C-640B) operating at 40 MHz repetition and depleted with a linearly polarized pulsed (1 ns) laser at 775 nm (Onefive Katana HP). In both nanoscopes, the power of the lasers was adjusted to obtain the best possible resolution in a given set of stained samples, and the imaging conditions were maintained within samples of the same experiment. The lateral resolution reached was 70–100 nm, which was empirically estimated by imaging isolated fluorescent molecules and measuring the FWHM of the obtained image (Supplementary Fig. S1).

### MPS evaluation: periodicity

The mean distance between βII-spectrin signals (“MPS periodicity”) was evaluated tracing a line starting at one bright spot and ending at the 5th consecutive to the first, using the ImageJ software (NIH). These measurements were divided by four to obtain the mean period of that segment. In expanded samples, the value obtained was further divided by the expansion factor of that experiment to obtain pre-expansion values. During image analysis, the experimenter was blinded to the group to which each image belonged.

### MPS evaluation: Assessment of organization and abundance

Regions of interest were divided in 1 μm × 1 μm sub-regions and analyzed using the *Gollum* software^22^, which is accessible at https://github.com/cibion-conicet/Gollum. The correlation value obtained corresponds to a Pearson’s correlation between the subregion and a modeled MPS period and is indicative of how preserved the periodic organization of the MPS is in that 1 μm section. The measurement was repeated along axons in the image with no overlapping, by an operator blind to the experimental conditions. To estimate MPS abundance, we first determined the correlation value that corresponded to a visible MPS (*threshold*), which corresponds to a value of 0.12 in Fig. 4b–d. MPS abundance is the proportion of sub regions with correlation values above the defined threshold MPS over the total number of segments evaluated (Fig. 3b). During image analysis, the experimenter was blinded to the group to which each image belonged.

### Statistical analyses and study design

Data are presented as mean ± SEM or mean ± SD, as indicated in the figure legends. Calculations were made using the statistical software InfoStat. Normality and homoscedasticity were proved for each group in order to apply the following parametric tests. When comparing two groups, Student’s t test (two-tailed) was used for statistical tests. When comparisons where made among three or more groups, one-way ANOVA was used; with Tukey post-hoc multiple comparisons, to find where the differences occurred. The *n* of each group is indicated in the figures or figure legends. Significance is indicated with an asterisk when p ≤ 0.05. The study design was not pre-registered. No randomization was performed to allocate subjects in the study. No sample size calculation was performed. We did not run tests to identify outliers and no data points were excluded from the analysis.

## Supplementary information

Supplementary information.
