# Supplementary material for: Quantitative expansion microscopy for the characterization of the spectrin periodic skeleton of axons using fluorescence microscopy
Source: Sci Rep. 2020 Feb 19;10:2917. doi: 10.1038/s41598-020-59856-w (PMC7031372; doi:10.1038/s41598-020-59856-w)
Supplement: Supplementary file 1 — Supplementary information. [file 41598_2020_59856_MOESM1_ESM.docx]

**Supplementary Information**

**Title**

Quantitative expansion microscopy for the characterization of the spectrin periodic skeleton of axons using fluorescence microscopy

**Authors**

Gaby F. Martinez, Nahir G. Gazal, Gonzalo Quassollo, Alan M. Szalai, Esther del Cid-Pellitero, Thomas M. Durcan, Edward A. Fon, Mariano Bisbal, Fernando D. Stefani and Nicolas Unsain

**Supplementary Figure 1: Determination of the minimum resolution needed to unequivocally reveal the periodic distribution of the MPS.**

**Supplementary Figure 2: Assessment of MPS periods in hippocampal neurons at 7 DIV in different locations to determine fidelity of the expansion.**

**Supplementary Figure 3: Examples of the immunolocalization of βII-spectrin in expanded dendrites and somas in hippocampal cultures.**

**Supplementary Figure 4. Alpha-tubulin immuno-detection before and after expansion in cell lines and neurites.**

**Supplementary Figure 1**


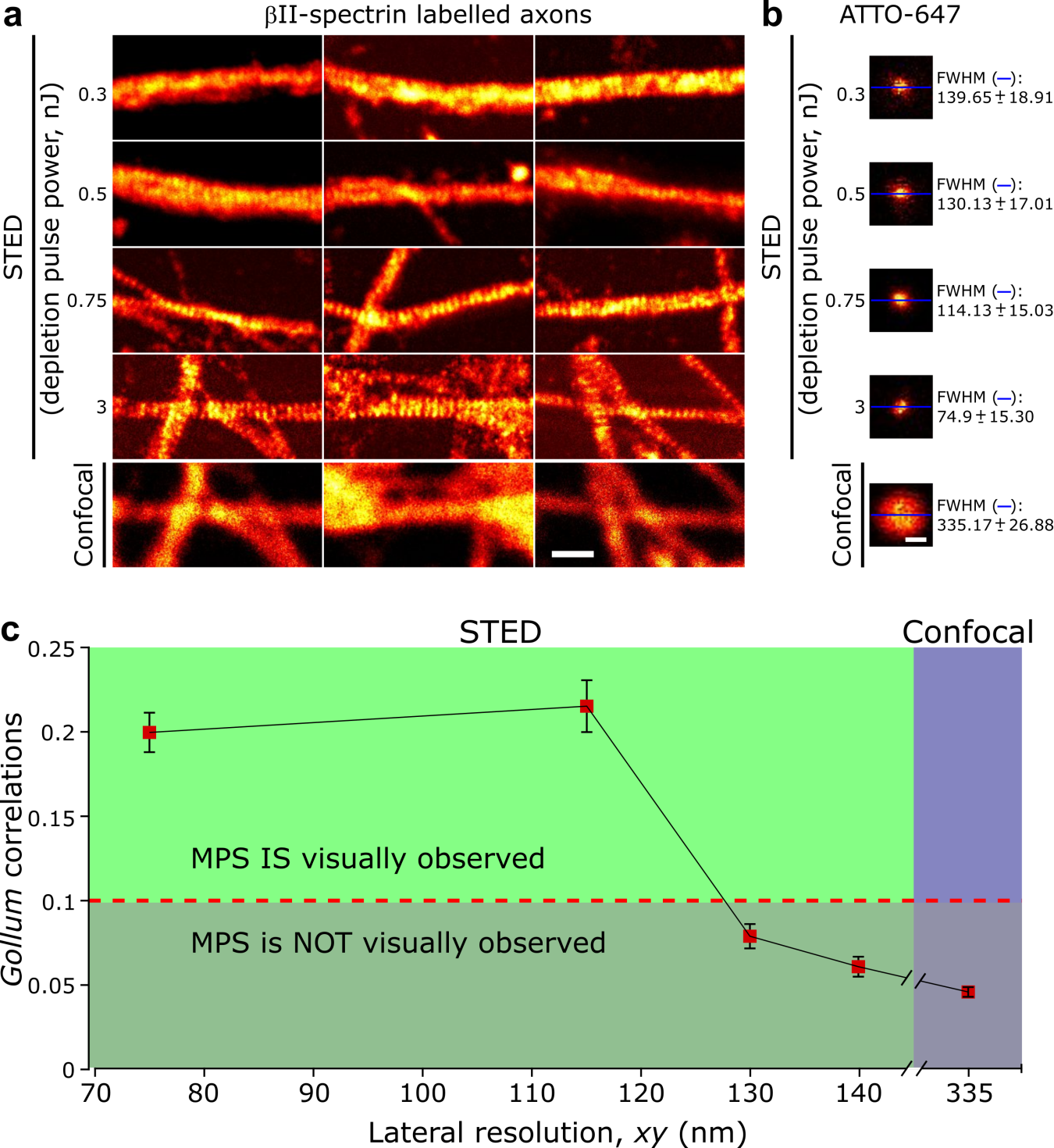


**Figure S1. Determination of the minimum resolution needed to unequivocally reveal the periodic distribution of the MPS.** We used a custom-built pulsed STED nanoscope to estimate the minimum lateral resolution to reveal the periodical distribution of βII-spectrin labeled with a primary antibody and an ATTO-647 labeled secondary antibody. We used hippocampal cultures grown for 40 days in vitro (DIV) and stained against βII-spectrin. In STED, the effective resolution obtained is proportional to the power of the depletion laser used. We first acquired images at different locations varying the power of the depletion laser, from 0.3 to 3 nJ per pulse. (**a**) By the naked eye, the periodicity clearly emerges between 0.5 and 0.75 nJ. Scale bar: 1 µm. (**b**) To define what is the effective resolution obtained at those powers, we measured the full width at half maximum (FWHM) of single molecule emitters. Scale bar: 200 nm. (**c**) To quantitatively address the emergence of 190 nm periods in the obtained images, we used *Gollum* (Barabas et al. 2017), to retrieve Pearson’s correlation values, and plotted them against the different lateral resolutions obtained for STED and confocal microscopes. We noticed that the 115 nm lateral resolution also signifies a marked increase in the correlation values obtained, providing a quantitative, unbiased threshold for revealing the MPS.

**Supplementary Figure 2**


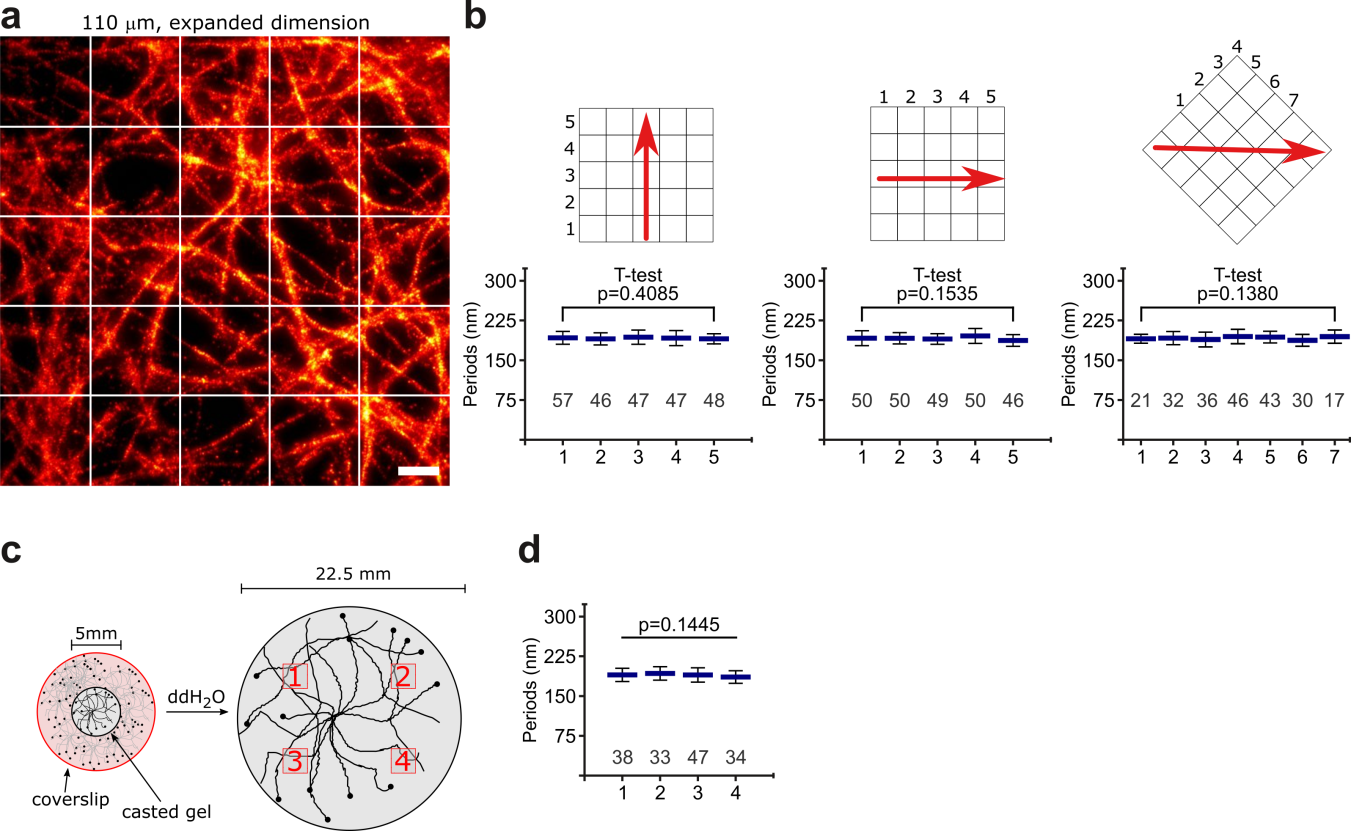


**Figure S2. Assessment of MPS periods in hippocampal neurons at 7 DIV in different locations to determine fidelity of the expansion.** (**a**) Maximum projection from an expanded stack used for the MPS periods measured in B. The overlaid grid was used to evaluate possible expansion aberrations that manifest in different directions. Scale bar: 10 µm. (**b**) Graphs showing the mean quantified periods in rows (first graph), columns (second graph) and a diagonal (third graph) from the stack shown in A. The x-axis was built by grouping the grids as shown in the top schemes. The number in each category is the number of periods measured in each category. Graphs show mean ± SD. (**c**) Scheme showing the regions in the expanded sample used to quantify the periods shown in **(d**). (**d**) Mean quantified periods in 50x50 µm areas, as shown in A. The x-axis was built by grouping the grids as shown in the top schemes. The number in each category is the number of periods measured in each category. Graph shows mean ± SD.

**Supplementary Figure 3**

**
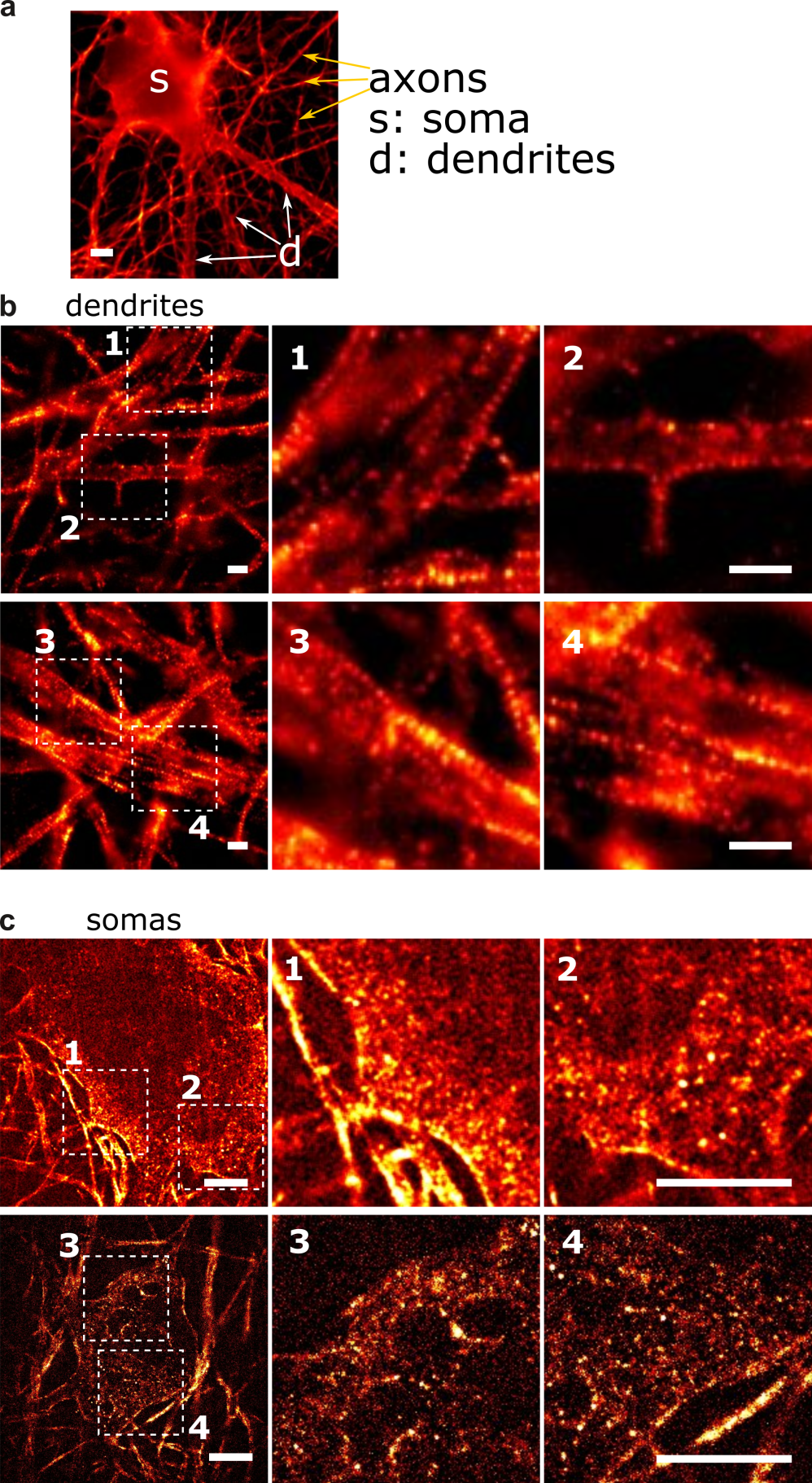
**

**Figure S3. Immuno-localization of βII-spectrin in dendrites and somas of expanded hippocampal cultures.** (**a**) βII-spectrin immuno-staining of hippocampal neurons in culture, in a pre-expanded sample. Note that dendrites and axons can be readily distinguished by caliber -dendrites being 5-10 times thicker than axons. Scale bar: 5 µm. (**b**) Single optical sections of dendrites in expanded samples, where the periodic localization of βII-spectrin was readily observed at the edges of the membrane. Scale bars: 1 µm. (**c**) Single optical sections of somas in expanded samples Examples, close to the coverslip, to show the localization of βII-spectrin in proximity to the inner side of the plasma membrane. Scale bars: 5 µm.

**Supplementary Figure 4**


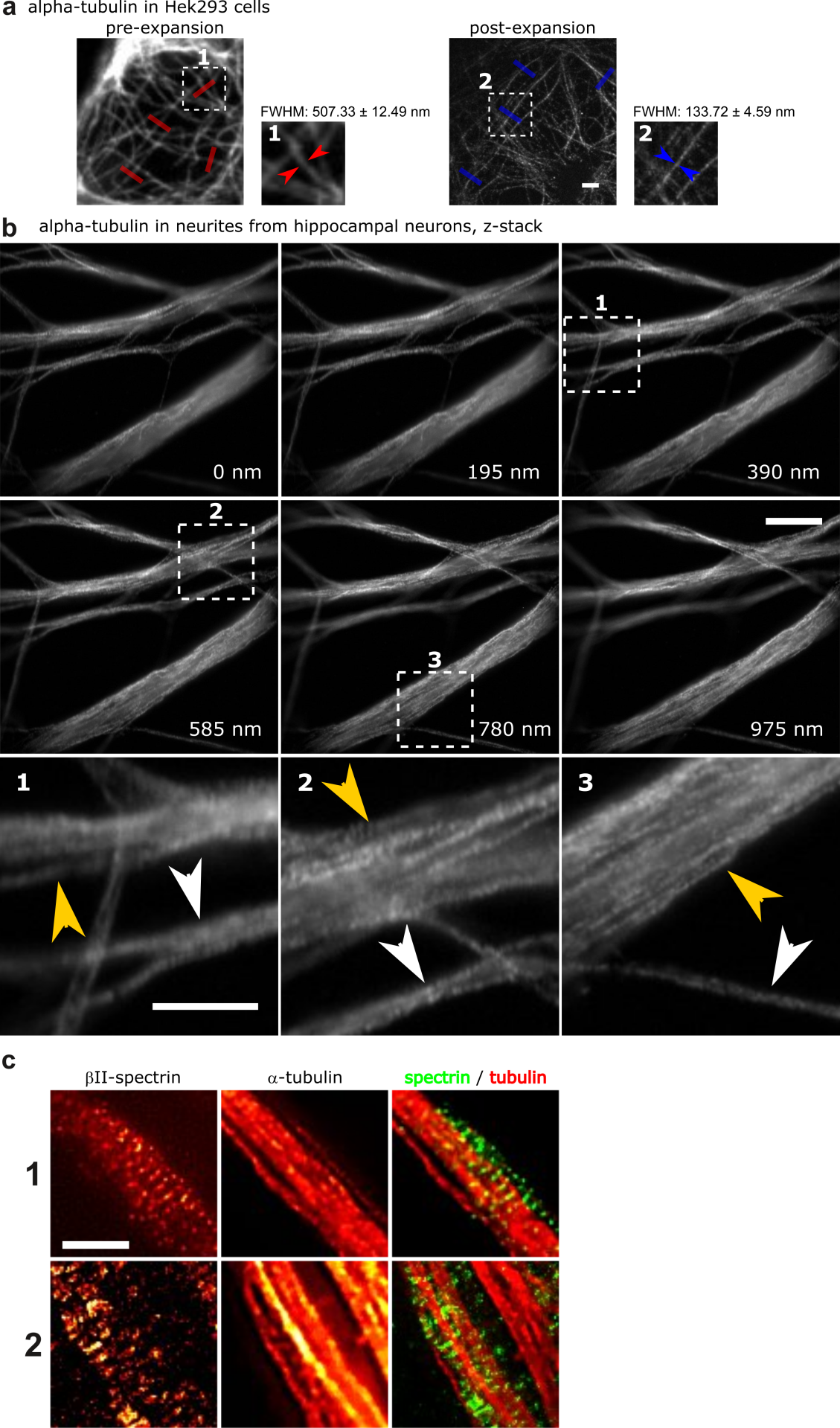


**Figure S4. Alpha-tubulin immuno-detection before and after expansion in cell lines and neurites.** (**a**) alpha-tubulin staining in HEK293 cells pre- (left) and post-expansion (right). The full width at half maximum (FWHM) of individual microtubules show that while before expansion their width is measured at 507.33 ± 12.49 nm, after expansion their width is 133.72 ± 4.59 nm. The FWHM was calculated using a plugin in ImageJ (FWHM line) using 3 pixels wide lines like the ones marked in the image in red and blue. Scale bar: 2 µm. (**b**) Post-expansion images of alpha-tubulin staining in hippocampal neurons at 7 DIV. The top 6 micrographs corresponds to a z-stack along the axial axis, an the number indicates the scaled increment along the series. In the inserts shown in the bottom row, note that while individual microtubular tracks can be distinguished in thick neurites (yellow arrowheads), they appear blurred in thin neurites (white arrowheads). Scale bars: 2 µm, 1 µm (inserts). (**c**) Additional examples of the co-immunostaining of βII-spectrin and α-tubulin, similar to the one shown in Figure 3, panel d. Scale bar: 1 µm.
